# Apple Fruit Recognition Algorithm Based on Multi-Spectral Dynamic Image Analysis

**DOI:** 10.3390/s19040949

**Published:** 2019-02-23

**Authors:** Juan Feng, Lihua Zeng, Long He

**Affiliations:** 1 Department of Agricultural and Biological Engineering, Pennsylvania State University, University Park, PA 16802, USA; juf618@psu.edu (J.F.); lzz153@psu.edu (L.Z.); 2 Department of Electronic Information Science, College of Information Science and Technology, Hebei Agricultural University, Baoding 071001, China; 3 Department of Electrical Engineering, College of Mechanical and Electrical Engineering, Hebei Agricultural University, Baoding 071001, China

**Keywords:** apple fruit, multi-spectral dynamic image, fruit recognition

## Abstract

The ability to accurately recognize fruit on trees is a critical step in robotic harvesting. Many researchers have investigated a variety of image analysis methods based on different imaging technologies for fruit recognition. However, challenges still occur in the implementation of this goal due to various factors, especially variable light and proximal color background. In this study, images with fruit were acquired with a Forward Looking Infrared (FLIR) camera based on the Multi-Spectral Dynamic Imaging (MSX) technology. In view of its imaging mechanism, the optimal timing and shooting angle for image acquisition were pre-analyzed to obtain the maximum contrast between fruit and background. An effective algorithm was developed for locking potential fruit regions, which was based on the pseudo-color and texture information from MSX images. The algorithm was applied to 506 training and 340 evaluating images, including a variety of fruit and complex backgrounds. Recognition precision and sensitivity of these complete fruit regions were both above 92%, and those of incomplete fruit regions were not lower than 72%. The average processing time for each image was less than 1 s. The results indicated that the developed algorithm based on MSX imaging was effective for fruit recognition and could be suggested as a potential method for the automation of orchard production.

## 1. Introduction

Apples are the second most valuable fruit grown in the United States after oranges [1]. Currently, hand picking is the only commercial harvesting method for fresh market apples, which is labor intensive and costly, accounting for more than 30% of production costs [2]. This intense seasonal labor demand is creating a significant risk for growers not having a sufficient supply of labor to conduct farm tasks. Harvesting, in particular, is threatened mostly by the uncertain availability of labor. The industry needs technological innovations, which can assist growers in maintaining a competitive position in the global marketplace. Robotic picking is one of the promising approaches to automate fruit harvesting. The main advantages of robotic picking are its ability to facilitate selective harvesting and its potential to reduce the dependence on the labor force. While research has been conducted on the production of harvesting robots, the commercialization of robotic harvesting has been hindered by technical and economic factors [3].

Accurate fruit recognition is one of the crucial steps necessary for the commercialization of robotic harvesting. Many studies have been conducted to recognize the fruit of horticultural crops automatically, such as sweet peppers, cucumbers, citrus, mangos, tomatoes, and apples [3,4,5,6,7,8]. However, challenges still remaining in the implementation of robotic harvesting in regard to fruit recognition. This is due to various factors, especially variable light, and proximal color background. Therefore, more attention has been focused on using different image acquisition devices and means to obtain useful images and favorable characteristic evidence to support the determination of fruit. Some researchers have utilized active imaging systems for fruit recognition: Liu et al. [9] developed a two-dimensional vision sensor system using two kinds of laser beams, to detect matured fruit based on the difference of laser reflectance on a plant, obtaining a recognition accuracy of 67%. Feng et al. [10] used a Time-of-Flight (ToF) camera to acquire multi-source images for the recognition of overlapped fruit, obtaining a fruit recognition rate of 83.67% to 94.22%. Nguyen et al. [11] used a Kinect sensor to acquire depth and color information for the detection and localization of red and bicolored apples, the processing time was below 1 s for a simultaneous detection of 20 apples. Other researchers have sought to create light-stable environments by adding light sources and shields: Song et al. [12] used a Xenon flashlight (with a light pulse to illuminate) to reduce the influence of ambient light on the images and a light shield to block direct sunlight. Gongal et al. [13] used a tunnel structure with a number of LED lights being installed inside to create a controlled, uniform lighting environment and also added capability for nighttime data collection, which achieved an identification accuracy of 79.8%. These past studies have mainly focused on the improvement of the hardware sensors for image acquisition, which could increase the overall cost, even though the recognition could be improved to some degree. These efforts, from a sensor system improvement perspective, are mainly aimed to eliminate the effect of sunlight variation. However, changes in sunlight can also help us to distinguish objects that have different responses in terms of thermal radiation.

With the development of visual sensor technology, an increasing number of object characteristics can be recognized using sensors. Besides using color, shape, and texture, the surface temperature variation of different objects under different conditions has also become a very important feature for object recognition. Thermal radiation is defined as the phenomenon of radiant electromagnetic waves due to the temperature of an object. All objects with a temperature above absolute zero can emit thermal radiation, and the higher the surface temperature, the more radiant the energy. Thermal imaging utilizes electromagnetic radiation emitted from an object and produces a pseudo image of the thermal distribution of the object [14]. In the pseudo images, different objects show in different colors, which could be potentially used for object identification in agricultural production. Recently, a number of applications utilizing thermal imaging have been reported. Raza et al. [15] combined thermal and visible light image data with depth information to remotely detect plants infected with the tomato powdery mildew fungus. Zhu et al. [16] used infrared thermal imaging technology to detect the temperature information of tomato and wheat during the incubation period following the introduction of inoculum. Satone et al. [17] assessed the surface of apples and subsurface defects with thermal images. García-Tejero et al. [18] and Wiriya-Alongkorn et al. [19] utilized thermal imaging to detect plant water stress, which could be an interesting tool for improving irrigation scheduling [18,19]. The studies above analyzed the difference of the plants with stress issues, rather than healthy (well-managed) plants. Some other studies have reported on the identification of different objects in an image using surface temperatures. For example, a few studies have worked on the recognition of citrus fruit from the tree canopy, including the appropriate time for acquiring fruit images, and integration of color image with image registration [20,21,22]. The image registration is an indispensable process, and the accuracy of image registration is one of the factors affecting recognition results. An innovative MSX technology, unlike traditional thermal superposition techniques, integrates infrared images with visible light images and highlights the texture characteristics of objects in infrared images without additional registration. The imaging results using this technology not only increase the evidence to support the fruit region, but also save the time in regard to image processing.

In this paper, MSX images were analyzed in different shooting situations to determine the best acquiring conditions (such as the optimal timing and angle range for acquiring images), helping to detecting the fruit regions in the image. An algorithm for recognizing apples on the tree canopy was developed. Regarding to pseudo-color characteristics of fruit region and noise characteristics in non-fruit region, a series of image pre-processing steps were carried out, including image segmentation based on color information, image denoising using morphological theory and small area removal strategies, labeling the effective regions, and restoration of color details. Following this, texture features of these effective regions were extracted and filtered to form the feature vector. A linear separable support vector machine was utilized to distinguish fruit regions from non-fruit regions. Finally, the three proposed measurement parameters, including precision, sensitivity, and relative error, were utilized to assess the effect of fruit recognition. The outcome from this study will be expected to provide guideline information for automated orchard production.

## 2. Materials and Methods

### 2.1. Imaging Acquisition System

**Figure 1:**
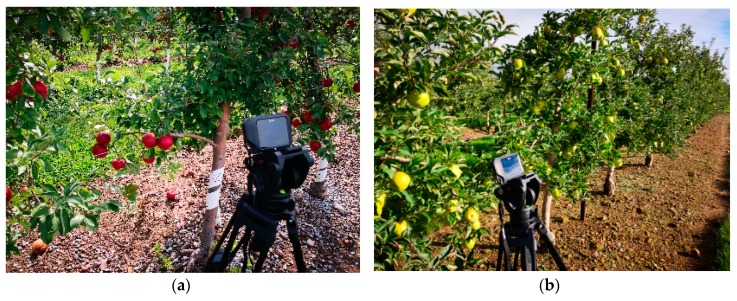
Illustration the imaging system placed in different scenes to acquire images: (**a**) Crimson Crisp apple trees; (**b**) Gold Rush apple trees.

A low-cost thermal camera (FLIR C3, FLIR Systems Inc., Wilsonville, OR, USA) was used to acquire images for fruit recognition (Figure 1). The camera had an operational temperature range from −10 °C to 150 °C with a spectral range from 7.5 μm to 14.0 µm an accuracy of ±2 °C of readings and a field of view of 41° × 31°. It can simultaneously capture three kinds of registration images, i.e., visible image, thermal image, and MSX image. These images were imported from a software (FLIR Tools+, FLIR Systems Inc., Wilsonville, OR, USA) and saved as JPG files with a spatial resolution of 400 × 300 pixels for computational efficiency. A total of 846 images were used in this study. Among them, 506 MSX images were chosen randomly as the training dataset, and the rest of 340 MSX images were used for evaluating the recognition effect. Image analysis was conducted with a computer (Dell G5 15, Dell Inc., Round Rock, TX, USA) with a CPU (Intel i7-8750H, Intel Inc., Santa Clara, CA, USA) running at 2.21GHz, 16GB of RAM. The image processing toolbox in MATLAB (Ver. R2018a, Mathworks Inc., Natick, MA, USA) was used to process images by running the developed algorithm.

### 2.2. Experimental Environments

The experiment was conducted at research orchards located in Penn State Fruit Research and Extension Center, Biglerville, Pennsylvania, USA. Two apple varieties were tested, i.e., ‘Crimson Crisp’ and ‘Gold Rush’. These trees were planted in a tall spindle structure, and were supported by a trellis system of wires and poles. Images were acquired from August to October, the distance between camera and tree canopy was 1 to 1.5 m. The number of fruit in each image ranged from 2 to 10. To test the thermal radiation of different parts on fruit tree responses to sunlight variation, some partially cloudy days with a wind breeze were chosen for our experiment. The thermal camera was placed at the bright side of the apple trees in the morning. We began to acquire images from 9 am, the temperature of the atmosphere was above 20 °C wind speed was around 3 mph, and average duration of cloud cover was 3 min. In all images, fruit regions with more than 60% occluded areas by branches and leaves were non-target fruit, and other fruit (including inter-fruiting occlusion) were defined as target fruit.

### 2.3. Fruit Tree Image Characteristics Analysis

#### 2.3.1. Image Characteristics of Different Apple Varieties

**Figure 2:**
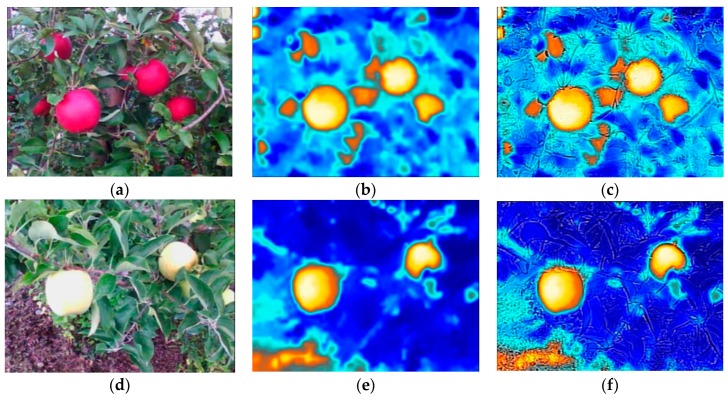
Illustration of images acquired by FLIR C3 camera (FLIR Systems Inc., Wilsonville, USA) in two different scenes: (**a**) RGB (Red, Green, Blue) image from part of a Crimson Crisp apple tree; (**b**) Thermal image for the targeted part of a Crimson Crisp apple tree; (**c**) MSX image for this part of a Crimson Crisp apple tree; (**d**) RGB image from part of a Gold Rush apple tree; (**e**) Thermal image for this part of a Gold Rush apple tree; (**f**) multi-spectral dynamic imaging (MSX) image for this part of a Gold Rush apple tree.

Different varieties of apples may show different colors through different stages of growth. Some fruit are easy to identify precisely from the image, while others are more difficult because they are masked with the similar color of the background or are occluded. Red apples (such as Crimson Crisp apple) provide a strong contrast to the background, and color could be used as an important feature to identify the fruit. While, for yellow apples (such as Gold Rush apple), especially in the early stages, the color is closer to green, the color would not be a sufficient characteristic to enable recognition of the fruit. Figure 2a,d shows RGB (Red, Green, Blue) images from different varieties of apple trees. However, thermal imaging is independent to the surface color, instead, it senses objects by detecting their emitted thermal radiation. In order to highlight the response of each part, the ‘Arctic’ was selected from color palettes to record the information. Figure 2b,e are the thermal images for the two selected varieties. In most of these thermal images, fruit regions appear in orange and show a large difference with other regions. We can also see that some background (such as the lower left corner of Figure 2e) shows a similar color to the fruit, which will interfere with the detection of the fruit by using color information alone. However, MSX images include detailed information as well as thermal radiation information, as shown in Figure 2c,f, the surface of fruit is smooth and the background is rough. The additional texture information will enhance recognition confidence of the fruit region.

#### 2.3.2. Image Characteristics with Different Camera Shot Angles

**Figure 3:**
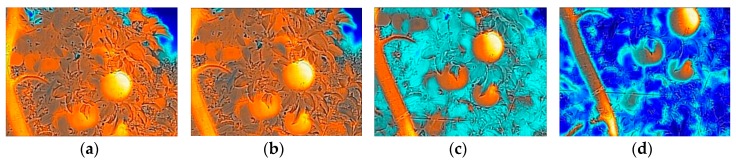
Partial fruit tree MSX images with different camera shot angels: (**a**) 0°; (**b**) −6°; (**c**) −12°; (**d**) −16°.

About 50% of the solar radiation energy is in the visible spectrum (wavelength of 0.4 to 0.76 µm), some in the infrared part (>0.76 μm) and the ultraviolet part (<0.4 μm). The thermal camera picks up the infrared spectrum. When the camera shooting angle (defined as the angle between ground level and the camera during image acquisition) is too high, solar radiation in the background will interfere with the display of fruit surface radiation in thermal images. A preliminary test was conducted by rotating the pan-tilt of the camera tripod counterclockwise with a step of 2 degrees to change the shooting angles. Some of MSX images shot from different angles are shown in Figure 3. The fruit begins to be identified in color, in contrast to the leaves and twigs surrounding them at the shooting angle of −12°. The color contrast is increasing until the shot angle is close to −16°. Therefore, the shooting angle of −16° was used in the following experiment to acquire images.

### 2.4. Fruit Recognition Algorithm

**Figure 4:**
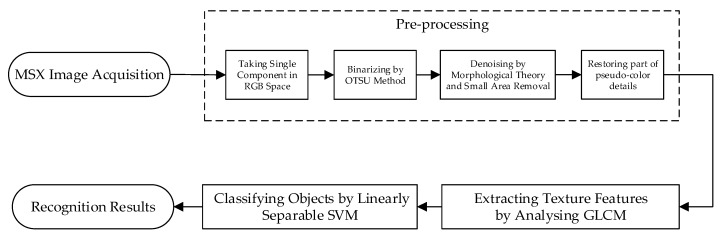
Overview of the automated fruit recognition algorithm.

A fruit recognition algorithm was developed in this study to be used with the MSX images. The proposed algorithm mainly consists of image pre-processing, texture features extraction, and object classification as shown in Figure 4.

#### 2.4.1. Pre-Processing

Pre-processing for these acquired images was conducted to preserve only the region of interest (such as orange representing a possible fruit region) by removing other color regions such as leaves, branches, or trunks, we could reduce the overall fruit recognition processing time. The pre-processing of MSX images consists of grayscale image processing by taking a single component in RGB space, binary processing through OTSU algorithm [23], morphological open operation, and small area removal method for de-noising, and restoring part of pseudo-color details.

More specifically, the value of each pixel in an MSX image is not directly reflected to that of the appeared color. Instead, these pixel values are used as the entry address of a table item in a color look-up table, to find the intensity values of R, G, and B used to display an image. Compared with a black-and-white grayscale image, the pseudo-color image enhances the image effect and enriches the image information. Considering that most of fruit are distributed in the orange region of an MSX image, which is close to the red component (one component in an RGB color space), the brightness value of a single red component is extracted by the component method, and the grayscale processing of MSX image is carried out.

In order to further highlight the difference between a target and background value, the grayscale image is binarized by OTSU algorithm (which is an image processing algorithm named after Nobuyuki Otsu). The noise in the binary image is analyzed, including some small objects with random distribution and different sizes. By using a morphological open operation, the disk-shaped structural elements are selected for filtering. Following this, the small area removal method is used for secondary filtering, with the main purpose being to remove unwanted objects with a slightly larger area. In this study, every connected region was labelled and the area was counted, the value of one tenth of the maximum connected area in the binary image was chosen as the threshold, and connection area larger than that was saved. Finally, the original pseudo-color information was given to the region with zero value to restore the details of the image, which can be used to further eliminate interference from the non-fruiting regions for post-processing.

#### 2.4.2. Texture Feature Extraction

After pre-processing, whole fruit, partial fruit, and non-fruit objects are possibly preserved in the pre-processed images. In order to retain only the fruit region (even when it is incomplete), some important features need to be extracted. Texture is an inherent property of the surface of an object, it is a kind of visual feature which does not depend on color or brightness, but reflects the homogeneity of an image. Therefore, texture has been used as an important characteristic for recognizing objects or regions of interest in an image. Haralick et al. [24] proposed 14 characteristic parameters for analyzing a gray-level co-occurrence matrix (GLCM). However, not all of these features are relevant, and there is a lot of redundancy in the computation. Bo et al. [25] analyzed the relationship between the parameters of GLCM and created a simplified choice of parameters in regard to texture calculation, three uncorrelated parameters, such as contrast, entropy, and correlation, were proved to be the best characteristics for recognition.

In addition to the target objects, there are still a large number of constant areas (all pixels in these areas are 0) in an image. If such data is used to calculate texture feature parameters, it will weaken the texture characteristics of the object. In order to better and faster represent the texture variation in a connected region, a cell array C(i,j) was created for different connected regions in different image (i stands for image sequence number, j stands for connected region number in an image). The data for each connected region was stored sequentially in the size of the minimum bounding rectangle in each cell of the cell array. That is, each cell in the cell array contained a connected object data, whose constant areas were as small as possible.

In the processing of texture feature extraction, the calculated step was two, and the angles of 0°, 45°, 90°, 135° for texture feature directions were used to generate the GLCM and to calculate the eigenvalues. The texture feature is sensitive to the direction, but different directions have little influence on it. Therefore, when quantifying the texture model, the average value of the eigenvalues obtained from the four directions can be used as the eigenvalues of the parameters in the model.

#### 2.4.3. Objects Classification

The concept of object classification is to construct a classification model based on existing data. The model can map some data in a database to a given category, which can be applied to data prediction. The support vector machine (SVM) is an effective method for classification, which derives from statistical learning and uses a supervised learning model for training. The learning strategy of SVM is to maximize the spacing, which can be formalized into a problem for solving convex quadratic programming [26]. After pre-processing, n features are extracted, q connected regions are selected as training samples, n×q feature parameters are obtained, and according to their categories, the class tag −1 or 1 of connected regions are assigned as input of two classes SVM predictive model, and then SVM classifier is obtained. By observing the characteristics, the extracted texture parameters are linearly separable and can be classified based on the decision classification function (as Equation (1)).

(1) $$f\left(x_{i}\right)=sign\left(\omega^{*}\cdot x_{i}+b^{*}\right)=\{\begin{array}{l} -1\;fruit\;region\\ +1\;non\;fruit\;region \end{array}$$

where $x_{i}\;$is the feature vector for the *i*th connected region, $\omega^{*}\;$is the optimized weight vector, and $b^{*}$ is the optimized bias value.

### 2.5. Measurable Parameters

To assess the accuracy of the proposed image processing algorithm, a few measurable parameters were defined. The true fruit regions in MSX images segmented by a manual method were compared with the fruit regions segmented by the proposed method. There were two types of regions inside the manual segmentation region, namely the correct segmentation region $S_{ic}$ and the leakage segmentation region $S_{il}$, and another two types of regions outside the manual segmentation region, namely the correct segmentation region $S_{oc}$ and the over-segmentation region $S_{oo}$. Three ratios were used to assess the accuracy of recognition using the above four defined regions, Equations (2)–(4) are the corresponding formulas.

(2) $$R_{P}=\frac{S_{ic}}{S_{ic}+S_{oo}}\;$$

(3) $$R_{S}=\frac{S_{ic}}{S_{ic}+S_{il}}$$

(4) $$R_{E}=\frac{S_{oo}+S_{il}}{S_{ic}+S_{il}}$$

$R_{P}$ is the recognition precision, and represents the proportion of the true fruit regions in the detected fruit regions; $R_{S}$ is the recognition sensitivity, defined as the ratio of the detected true fruit regions to the artificially detected fruit regions; and $R_{E}$ is the recognition relative error, which is calculated as the ratio of the detected false fruit regions to the artificially detected fruit regions. These parameters are helpful to judge the centroid position and size of the fruit regions, which is critical information for positioning the manipulator for robotic picking.

## 3. Results and Discussion

### 3.1. Image Effects Using Different Light Stimulation

**Figure 5:**
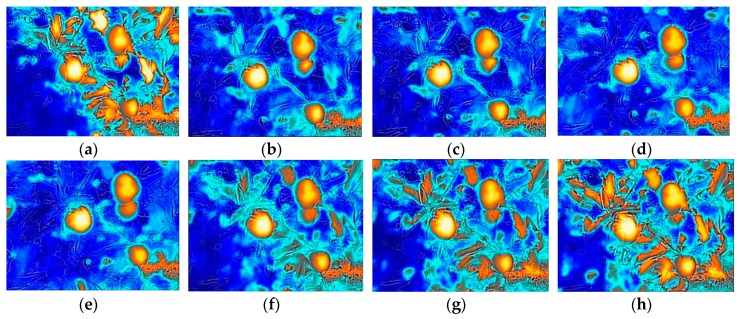
MSX images for a scene of fruit tree over a period of time during the cloud covering sun on 28 September 2018 (a typical partially-cloudy day). (**a**) 9:14:03; (**b**) 9:15:32; (**c**) 9:15:37; (**d**) 9:16:32; (**e**) 9:17:42; (**f**) 9:18:51; (**g**) 9:19:21; (**h**) 9:19:45.

**Figure 6:**
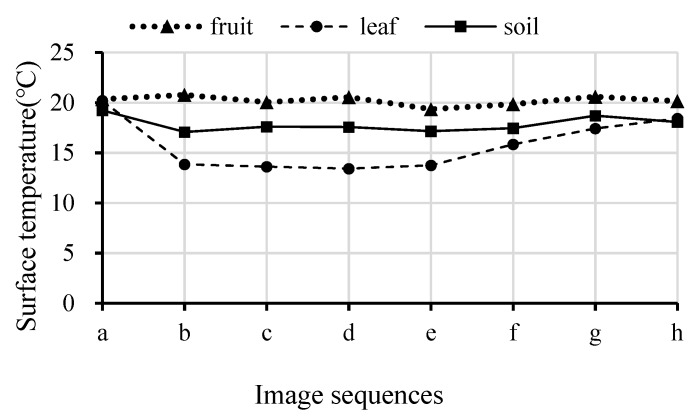
The surface temperature change in the fruit, leaf, and soil corresponding to the acquired images in Figure 5.

Theoretically, each part exposed on the fruit tree receives roughly the same amount of radiation in the atmosphere. While the surface temperature may different for different objects due to their natural specifications, for example, fruit contains more water than branches and leaves, and water absorbs and releases heat more slowly. Therefore, when the same amount of light energy is changed, each part of a fruit tree will have different surface temperature and different thermal response. Figure 5 shows a series of thermal response images over time for a targeted section of a fruit tree during a period of shade. The surface temperature of each object was imported from FLIR Tools+. Figure 6 shows the surface temperature of the three types of objects within the image over time corresponding to Figure 5. The rapid release of heat energy caused the leaf temperature to change quickly and obviously, while the influence was slight for the fruit. The change in surface temperature for leaf and fruit generated a big difference between the two objects. When the surface temperature difference between the two exceeded 6 °C, a large contrast appeared in the image (Figure 5b–d). In the image, there was a patch of soil in the background at the lower right corner. The surface temperature of soil only had slight change over the image acquisition period, and it was close to the surface temperature of fruit.

### 3.2. Recognition Results on Different Images

The following subsections describe the performance of the proposed fruit recognition algorithm.

#### 3.2.1. Image Pre-Processing Results

**Figure 7:**
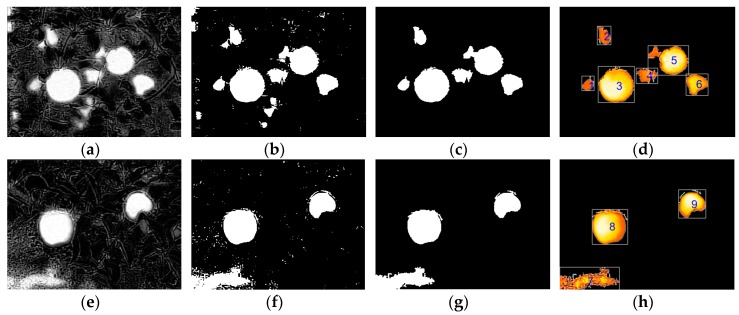
Illustration of different steps of images pre-processing in two examples showed in Figure 3: (**a**) Figure 3c grayscale processing; (**b**) Figure 3c binary processing; (**c**) Figure 3c denoising; (**d**) Figure 3c restoring and region labeling; (**e**) Figure 3f grayscale processing; (**f**) Figure 3f binary processing; (**g**) Figure 3f denoising; (**h**) Figure 3f restoring and region labeling.

Figure 7 shows the pre-processing results of fruit images under different scenes. The acquired fruit images were not affected by the color of the fruit after grayscale treatment (Figure 7a,e). When the camera shot angle and time were in the above conditions, the background had little influence on fruit recognition. Morphological theory and the minimum area denoising method can eliminate most of the interference which affects the recognition results (Figure 7c,g). By restoring the details of the retained regions (Figure 7d,h), it is possible to further remove objects that do not differ in terms of surface thermal radiation. The texture features are obviously different, such as the seventh labelled region (Figure 7h).

**Figure 8:**
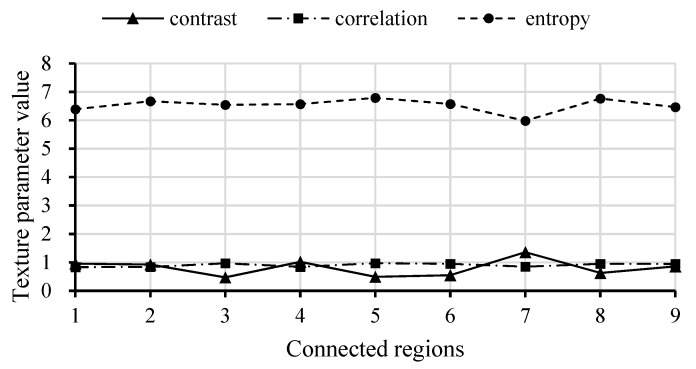
Three texture feature data graphs of nine labelled regions in two images.

The contrast, entropy and correlation of the nine labelled regions were calculated, the results are shown in Figure 8. In the figure, all the connected regions are fruit regions except the seventh region, which is a non-fruit region. Because the contrast value significantly increased while the entropy value decreased in the non-fruit region, this changing pattern was very different for the two values in the fruit region. Therefore, the contrast and entropy can be used as a set of separable indicators for two classes of objects. Among them, the contrast can reflect the depth of a texture groove, and the larger the contrast, the deeper the groove. Compared with the smooth surface of fruit, the groove effect of soil was more obvious. Therefore, the entropy represents the uniformity or randomness of content distribution, and the higher value, the more heterogeneous the distribution in the image. Because each labelled region has a portion of darkness, the region of the soil appears to be more homogeneous than the region of the fruit. Therefore, contrast and entropy were chosen as the texture features to be used in object classification.

#### 3.2.2. Assessment of Recognition Results

**Figure 9:**
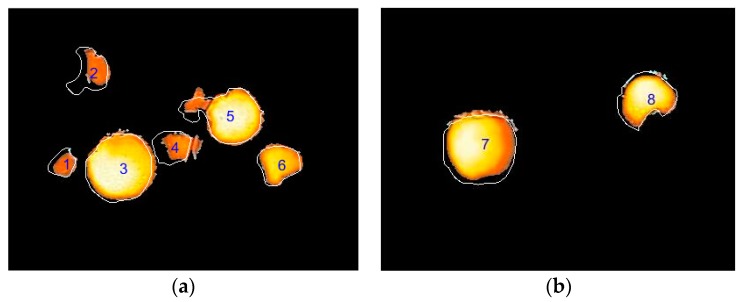
The fruit identification results of two examples with the proposed recognition algorithm and manual method. (White lines represent the contours of the fruit marked manually).

In Figure 9, the orange areas are the fruit regions obtained using the proposed algorithm, which were compared with the white lines represented the contours of the fruit by a manual method. Among all regions, fruit regions 3, 6, 7, and 8 achieved the ideal recognition effect, although these fruit contours were slightly smaller than the actual size. The main reason was that the fruit were mostly exposed to the outside of the tree canopy, the edge temperature was slightly lower than the front of the fruit due to the deviation from direct sunlight. Regions 1, 2, and 4 were seriously covered by branches and leaves, and there were significant differences in fruit surface temperature. Partial regions were misidentified as non-fruit regions because of the lower temperature. Region 5 contained two partially overlapped apples, the front fruit was ideally recognized, but the rear fruit was only partially recognized. Following this, Equations (2)–(4) were applied to each image to calculate three measurable parameters. Table 1 shows the fruit regions count, recognition precision, sensitivity and relative error for 340 MSX images. The fruit regions included the complete fruit regions (such as region 3 and region 7 in Figure 9) and the incomplete fruit regions. Incomplete fruit regions included the fruit overlapping (like region 5 in Figure 9) and foliage shading (like region 1, 2, 4, 6, and 8 in Figure 9). As can be seen form the Table 1, the average recognition sensitivity and accuracy of the complete fruit regions were 95.74% and 92.69%, and these of the incomplete fruit regions were 87.50% and 72.96%, separately. These show that the whole method was more sensitive and effective for fruit region recognition. Even though the average relative error in the incomplete fruit regions was a little large, the success rate of the recognition method using the low-cost imaging acquisition system is very encouraging.

### 3.3. Time Efficiency Analysis

To increase overall harvesting productivity, the time devoted to automatic recognition of fruit would be short. Table 2 lists the average execution times for the different processing steps for fruit recognition. The most time consumption task was the image pre-process, which took more than 90% of the overall time. Overall, the average process time for an image was less than 1 s in an entire picking cycle. The results show that the proposed algorithm is suitable for integration in a harvesting robot.

### 3.4. Discussion

From the results above, the proposed algorithm for detecting the fruit with MSX images had fairly high accuracy, accounting for 95.74% for the complete fruit region and 87.50% for the incomplete fruit region Overall, the accuracy of fruit recognition in images was about 91.62%, which is comparable with some previous studies introduced earlier [9,10,11,12,13]. Specifically, the fruit detection in this study is mainly for robotic harvesting of fruit, and our method could provide accurate fruit region for harvesting with spatial information. Therefore, compared to the precision and recall of fruit number recognition in some previous studies, the precision and sensitivity of the specific fruit region is more practical for fruit harvesting.

Typically, during robotic harvesting, fruit images are acquired and processed prior to the robotic arm executing [11]. In this study, the algorithm took about 740 ms to process an image, which is about 120 ms for recognizing one fruit region with the average number of six pieces of fruit in an image. The movement of the robotic arm to reach a fruit in a harvesting cycle normally takes much longer than 120 ms, and the speed we achieved for fruit detection could be sufficient for the harvesting process. As we can see, the pre-processing step takes up a large proportion of the total time in our algorithm, and it could be potentially improved by using a multi-thread acceleration method in the future.

In addition, MSX images are sensitive to both surface texture and thermal radiation of objects. Therefore, more features are needed to increase the evidence to support the fruit region. However, different parts of a fruit tree that appear in MSX images also depends on some shot conditions. Among them, shot angle and shot timing are the main considerations. The effect of solar radiation on the image can be effectively suppressed by shooting fruit trees from an overhead camera. Through the temporary process of the sun being covered by cloud, the fruit, branches, and leaves can appear with a larger color contrast in the image. Therefore, knowing that the best time to shoot is not limited to a certain time period, as long as the surface temperature difference of each part reaches a certain amount by covering, a better image effect can be achieved.

## 4. Conclusions

This paper analyzed fruit recognition performance using a novel kind of image (MSX image). When the camera shooting angle was close to −16° the maximum contrast between target and background was obtained in an MSX image. Due to the difference of capability to absorb and release heat, changing sunlight on a fruit affected the surface temperature more slowly than neighboring leaves. It indicated that fruit and leaf can be identified easily when the temperature difference between them was higher than 6 °C. Therefore, it is possible to adjust direct light intensity by covering the targeted tree canopy. Determination of covering duration, covering material, and covering area will be our researched next.

An effective algorithm was developed to detect fruit in the MSX images. The red component of input images was chosen as it highlighted the characteristic of the target. Morphological theory and small area removal strategy effectively removed non-target regions in binary images; the texture characteristics were extracted to enhance the support judgment of the target regions; and the final results were obtained by the linear separable SVM. During the processing period, most fruit regions can be detected, and the feature vectors used in the post-processing are few, so it is faster to use a simple classification model.

The average processing time for the entire algorithm is 740.04 ms, the recognition precision and sensitivity of complete fruit regions were above 92%, and those of incomplete fruit regions were not lower than 72%. In order to improve the recognition of the incomplete region, multi-view image fusion can be considered. In addition, the picking strategy can be designed to start from the outermost layer of fruit, layer-by-layer recognition and picking, which can also effectively reduce the possibility of overlapped fruit.

## Figures and Tables

**Table 1:** The results of measurable parameters calculation.

Parameters	Complete Fruit Regions	Incomplete Fruit Regions
No. of regions	759	1012
Average recognition precision	95.74%	87.50%
Average sensitivity	92.69%	72.96%
Average relative error	8.68%	37.04%

**Table 2:** The average execution times for the different processing steps.

Step	Processing Time (ms)
Image Pre-processing	725.63
Texture features extraction	13.32
Objects classification	1.09
total	740.04
